# Understanding Exercise-Induced Hypoalgesia: An Umbrella Review of Scientific Evidence and Qualitative Content Analysis

**DOI:** 10.3390/medicina61030401

**Published:** 2025-02-25

**Authors:** Mario González-Iglesias, Alexis Martínez-Benito, Javier Andrés López-Vidal, Alberto Melis-Romeu, Daniel Jacobo Gómez-Rabadán, Álvaro Reina-Varona, Silvia Di-Bonaventura, Roy La Touche, José Fierro-Marrero

**Affiliations:** 1 Centro Superior de Estudios Universitarios La Salle, Universidad Autónoma de Madrid, 28049 Madrid, Spain; mariogonzalez.pt@gmail.com (M.G.-I.); alexis.martinezben@gmail.com (A.M.-B.); javlopezvid@gmail.com (J.A.L.-V.); melisllauri@gmail.com (A.M.-R.); danielgomezrabadan@gmail.com (D.J.G.-R.); alvaroreina93@gmail.com (Á.R.-V.); jose.fierromarrero@yahoo.com (J.F.-M.); 2 International Doctorate School, Department of Physical Therapy, Occupational Therapy, Rehabilitation and Physical Medicine, Universidad Rey Juan Carlos, 28933 Alcorcón, Spain; 3 Motion in Brains Research Group, Centro Superior de Estudios Universitarios La Salle, Universidad Autónoma de Madrid, 28049 Madrid, Spain; 4 Department of Physical Therapy, Occupational Therapy, Rehabilitation and Physical Medicine, Universidad Rey Juan Carlos, 28032 Madrid, Spain; silvia.dibonaventura@urjc.es; 5 Cognitive Neuroscience, Pain and Rehabilitation Research Group (NECODOR), Faculty of Health Sciences, Universidad Rey Juan Carlos, 28032 Madrid, Spain; 6 Grupo de Investigación Clínico-Docente sobre Ciencias de la Rehabilitación (INDOCLIN), Centro Superior de Estudios Universitarios La Salle, 28023 Madrid, Spain; 7 Instituto de Dolor Craneofacial y Neuromusculoesquelético (INDCRAN), 28008 Madrid, Spain

**Keywords:** exercise, exercise-induced hypoalgesia, pain threshold, healthy volunteers, musculoskeletal pain, information dissemination, social media, X

## Abstract

*Background and Objectives*: Exercise-induced hypoalgesia (EIH) is a topic of interest in the scientific community. This umbrella review aimed to analyze EIH research and compare it with public dissemination on X. *Materials and Methods*: We selected relevant EIH reviews that included a healthy population or patients with pain and studied exercise interventions. A systematic literature search was carried out in PubMed, Web of Science, SciELO, PEDro, and Google Scholar, employing the Population, Intervention, Comparison, and Outcome strategy. Data were extracted and summarized, and methodological quality was assessed with the Quality Assessment Scale for Systematic Reviews, and risk of bias with the Risk of Bias in Systematic Reviews tool. The Physical Activity Guidelines Advisor Committee was employed for evidence synthesis. Simultaneously, advanced X website searches gathered EIH-related content for analysis. Information from posts on X was qualitatively analyzed and contrasted with evidence in the literature. *Results*: We included nine systematic reviews and 17 narrative reviews. Systematic reviews presented high methodological quality. However, half had low risk of bias, while the other half presented high risk of bias. The EIH in healthy participants was controversial for some exercise modalities, such as aerobic exercise, and the influence of psychological variables. Modalities, such as isotonic resistance exercise, showed favorable effects on hypoalgesia. However, in patients with musculoskeletal pain, different exercise modalities did not generate EIH. X analysis unveiled a considerable representation of science-related content, although with prevalent misinterpretations of scientific evidence. *Conclusions*: EIH has been extensively studied, yet the certainty of evidence remains limited. While some exercise modalities demonstrate hypoalgesic effects in asymptomatic individuals, these effects remain unverified in patients with musculoskeletal pain. Moreover, the analysis of social media content highlights frequent misinterpretations of scientific evidence, particularly conflating hypoalgesia with analgesia. This underscores the need for more precise, evidence-based communication on social media platforms.

## 1. Introduction

Pain is the primary motivation for patients to seek medical attention [1]. Whereas acute pain is a symptom that aids in the healing of tissues, chronic pain is a condition that no longer serves a protective role and impairs an individual’s functionality and quality of life [2]. Persistent pain affects more than 20% of the European population, with approximately 9% experiencing pain on a daily basis [3]. Chronic pain is more prevalent among women, unemployed individuals with previous work experience, individuals with lower education levels, those living in poverty, and people residing in rural areas [3,4]. The occurrence of chronic pain rises with age, with a prevalence of 14.3% among individuals aged 18–25, and a staggering 62% among those older than 75 years [5].

Mounting evidence suggests that exercise, defined as organized physical activity, is a highly effective therapeutic approach for addressing chronic pain-related conditions. Exercise possesses several advantageous qualities, such as accessibility, cost-effectiveness, and the potential for beneficial outcomes [6,7]. Regular engagement in exercise has demonstrated the ability to enhance cardiorespiratory function, alleviate pain, and contribute to improved mental well-being [8,9,10,11,12].

Exercise-induced hypoalgesia (EIH) refers to the phenomenon in which pain sensitivity decreases after engaging in exercise [13]. EIH is usually quantified by applying a painful stimulus to the body before and after exercise and measuring changes in pain sensitivity, such as increased thresholds or decreased pain intensity, to a standardized painful stimulus. This phenomenon was first studied by Black et al. (1979) [14], who conducted a case report in which long-distance running increased pain sensitivity thresholds. Since then, EIH has been investigated in both healthy individuals and in those with musculoskeletal pain. Current evidence suggests a consistent EIH response in healthy individuals through various exercise modalities. However, in individuals with musculoskeletal pain, EIH presents higher variability depending on the type of exercise, pain condition, and pain location.

Social media provides large amounts of information in an accessible way. Among the numerous social platforms, X stands out as a highly popular microblogging platform known for its accessibility and effective data retrieval [15,16]. X encompasses a diverse range of information shared by both patients and healthcare professionals. Patients can express their perception of their medical conditions [17,18], and professionals can disseminate and access information relevant to health-related issues [19]. Given these features, we believe X is a useful social media platform for conducting a qualitative study to evaluate user’s knowledge about EIH.

Although several reviews examining the impact of exercise on pain sensitivity have been published [20,21,22,23], to our knowledge, there is currently no comprehensive umbrella review that consolidates the information from these existing reviews.

This umbrella review aims to summarize available evidence concerning the EIH phenomenon. Studies were selected following a precise inclusion and exclusion criteria. The Physical Activity Guidelines Advisor Committee (PAGAC) was employed for robust evidence synthesis. Concurrently, a secondary objective was to juxtapose the present scientific knowledge against the public’s perceptions expressed on X. This involved the use of search strategies on X’s website, data extraction, and a content analysis, all conducted while adhering to strict ethical standards.

The study aims to draw a comparison between the scientific evidence surrounding the concept of EIH and the public’s understanding of this concept and its related aspects, as reflected through a qualitative content analysis on the X platform.

## 2. Materials and Methods

The study was reported in accordance with the Preferred Reporting Items for Systematic Reviews and Meta-Analyses (PRISMA) statement [24]. The protocol of this umbrella review was established prior to conducting it and was registered with the International Prospective Register for Systematic Reviews, PROSPERO (CRD42023378440).

### 2.1. Selection Criteria

The following inclusion and exclusion criteria were based on the Population, Intervention, Comparison, and Outcome strategy.

#### 2.1.1. Population

Reviews should include healthy individuals or patients with any type of pain.

#### 2.1.2. Intervention

Reviews should explore any type of exercise, defined as “any intervention that involves active movement or voluntary contraction by the participant”. Reviews were excluded if they explored exercise in combination with other types of interventions.

#### 2.1.3. Comparator

Reviews including minimal intervention or any other exercise modality.

#### 2.1.4. Outcome Measures

Reviews should address quantitative sensory testing. Eligible outcome measures included pressure pain threshold (PPT), pressure pain tolerance threshold, heat pain threshold, heat pain tolerance threshold, cold pain threshold, cold pain tolerance threshold, conditioned pain modulation, and temporal summation (wind-up phenomenon). Additionally, reviews could explore these variables in terms of effectiveness, its underlying action mechanisms, and the influence of psychological factors.

#### 2.1.5. Study Design

Systematic reviews, with or without meta-analyses, and narrative reviews were eligible for inclusion. The inclusion of both types of reviews allowed for a comprehensive understanding of EIH, with systematic reviews providing robust, evidence-based findings, while narrative reviews offered a broader interpretation and discussion of the findings.

### 2.2. Data Sources and Searches

Systematic searches were conducted in MEDLINE (PubMed), Google Scholar, WOS, SciELO, and PEDro from February 2023 to October 2024. The search strategy was built and adapted for each database. No restrictions on date or language were applied. Search equations are described in Supplementary Material S1.

### 2.3. Selection Process

Two reviewers employed the same search strategy. Duplicate registries were detected with Rayyan.ai [25] and removed manually. Title–abstract screening and full-text eligibility were performed manually and independently by reviewers following the selection criteria. Disagreements were resolved by a third reviewer.

### 2.4. Data Extraction

The relevant information from the included studies was extracted by two independent reviewers and comprised authors, date of publication, design, number of studies included, aims, population type, age, sex, interventions, control comparator, outcome measures, number of studies included in meta-analyses, and results obtained.

### 2.5. Methodological Quality and Risk of Bias Assessment

Two independent reviewers assessed the methodological quality and risk of bias of the included reviews. The methodological quality of the systematic reviews was assessed with the Quality Assessment Scale for Systematic Reviews developed by Barton et al. (2008) [26]. The scale presents 13 items that can be answered with “No”, “In Part”, or “Yes”, scoring 0, 1, or 2 points, respectively. Total methodological quality ranges from 0 to 26 points, 26 being the highest quality achievable. This tool presents validity and moderate to good inter-rater reliability [26]. Reviews that obtained a total score greater than 20 points were considered to have high methodological quality [26].

Risk of bias was evaluated with the Risk of Bias in Systematic Reviews tool [27]. This tool assesses quality throughout 3 phases: relevance assessment; identification of biases in 4 domains (eligibility criteria, identification methods, data extraction, and data synthesis); and judgement of overall risk of bias [27]. Risk of bias judgements across domains were categorized as “low risk of bias”, “high risk of bias”, or “unclear”.

Methodological quality of narrative reviews was evaluated with the Scale for the Assessment of Narrative Review Articles (SANRA) [28]. This scale evaluates 6 items exploring justification, aims, literature search, referencing, scientific reasoning, and data presentation. Items can be scored with 0, 1, or 2 points, making a total possible score of 0 to 12, in which 12 indicates the maximum methodological quality achievable. Reviews with 8 or more points were considered to have high methodological quality. This scale has proven to be valid and reliable, with sufficient internal consistency and inter-rater reliability [28].

Interrater agreement was assessed using Cohen’s kappa coefficient. The following classification was employed to interpret the strength of the agreement based on the Kappa value: poor (<0.00), slight (0.01–0.20), fair (0.21–0.40), moderate (0.41–0.60), substantial (0.61–0.80), and almost perfect (0.81–1.00) [29].

### 2.6. Evidence Synthesis

Evidence synthesis was performed with the PAGAC for assessing the level of evidence across studies for every population, outcome measure, and exercise modality or psychological variable association. Findings across systematic reviews were evaluated according to 5 items: (1) applicability of results based on study populations, exposures, and outcomes; (2) generalizability to the target population; (3) risk of bias or study limitations; (4) quantity and consistency of the results obtained across the outcome of interest; (5) magnitude and precision of effect.

Items were assigned a level of certainty of evidence, categorized as “strong”, “moderate”, “limited”, or “not assignable”. These items were examined by 2 different evaluators, and the results of each were compared and agreed upon by a third evaluator [30].

### 2.7. X Searches and Inclusion Criteria

Nine advanced search strategies were used on X’s website to compile the posts. The search was conducted on 15–22 November 2024. Searches were conducted with English and Spanish terms. No language nor time restrictions were applied.

### 2.8. Data Extraction from Posts

Researchers extracted the following information concerning both descriptive data and content information: user’s username, user’s profession and account type (i.e., physiotherapist, physician, chiropractic, sports professionals, exercise physiologist, disseminations accounts, institutions, other healthcare professionals, other non-healthcare professionals, and non-identifiable), publication date, the interactions of posts (reposts, likes, and replies), whether it belonged to a thread, and the addition of supplementary materials, such as images, other posts, or links to scientific articles, videos, blogs, etc.

### 2.9. Posts Content Analysis

Content analysis was performed by 2 independent reviewers, classifying the information into 6 codes: (1) “scientific-related content”, which involved posts that were a synthesis of scientific data without specific citations. It involved the discussion and dissemination of scientific findings in a broad approach; (2) “scientific quotes”, in which posts provided scientific information along with explicit references to the sources; (3) “personal appreciation”, in which opinions or personal perspectives without scientific background were expressed; (4) “advertising or publicity”, referring to posts that aimed to promote or disseminate a particular service, event, or product, often pursued to generate interest or encourage participation; (5) “misinterpretation of scientific evidence” was applied when posts misrepresent scientific evidence, leading to inaccurate or misleading information; and (6) “non-analyzable content”, if posts did not contribute any meaningful information to the topic of investigation.

Each post was assigned to a single code. Prior to classification, both reviewers verified the code criteria with a third investigator to achieve a higher concordance rate. For those posts where there was no agreement, a third reviewer decided the final code assigned.

As part of the analysis, the posts were classified into 5 distinct themes, considering the main message and underlying significance. The themes involved the following: (1) effect of exercise (both hypoalgesia and hyperalgesia); (2) underlying neurophysiological mechanisms; (3) exercise modalities and prescription variables; (4) location of effect; and (5) psychosocial factors involved in EIH.

Two other independent reviewers associated each post to a theme and to a subtheme to determine which contents were the most frequent. The same post could be assigned to more than 1 theme and to a maximum of 2 subthemes. For those posts in which there was no agreement between the investigators, a third person assigned the subthemes. Posts corresponding to codes 4 and 6 did not provide any useful content to analyze; therefore, they were not assigned any theme or subtheme.

### 2.10. Ethical Considerations

To protect the privacy of X users, this research collected only those posts that were useful for the purpose of the study. In addition, any personal information that could help identify the authors, such as the username, was removed.

## 3. Results

### 3.1. Review Selection Process

**Figure 1:**
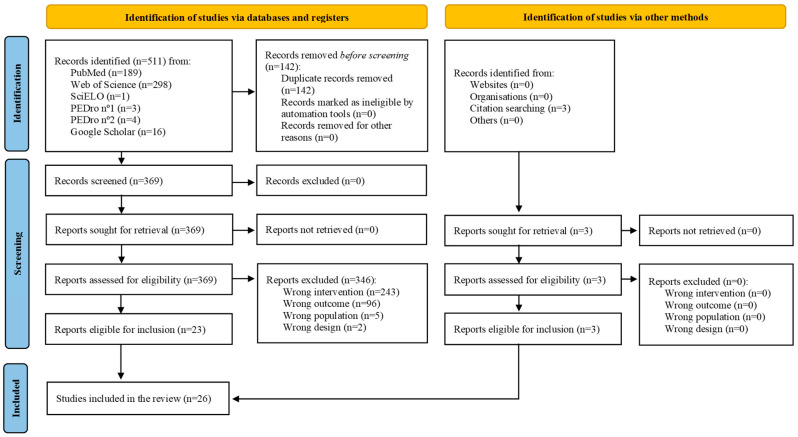
Flowchart diagram of studies’ selection process.

Twenty-six articles were ultimately included: five were systematic reviews with meta-analysis [20,21,22,23,31], four were systematic reviews [32,33,34,35], and 17 were narrative reviews [13,36,37,38,39,40,41,42,43,44,45,46,47,48,49,50,51]. Additional information is included in Figure 1.

### 3.2. Review Data Extraction

Systematic reviews and systematic reviews with meta-analysis included a total of 3913 individuals, of whom 1438 were male and 2475 were female. A total of 1088 individuals included in the exercise group were healthy, and 1559 presented any type of musculoskeletal pain. The mean age range of the healthy individuals included was 19 [52] to 64 years [53] and 20.4 [54] to 69 years [55,56] among patients with musculoskeletal pain. Additional information is provided in Table 1 and Table 2.

### 3.3. Methodological Quality and Risk of Bias Assessment

The nine systematic reviews were of average methodological quality (22.67 ± 1.73 out of 26 maximum points) in the Quality Assessment Scale for Systematic Reviews. The minimum score obtained was 20 [21], and the maximum was 26 points [20]. Inter-rater agreement was almost perfect (κ = 0.859). See Table 3 for more information.

The risk of bias assessment revealed four (44.44%) systematic reviews with low risk of bias [20,23,32,35] and five (55.56%) with high risk of bias [21,22,31,33,34]. Inter-rater agreement was perfect (κ = 1). See Table 4 for more information.

The methodological quality of the 17 narrative reviews were of average methodological quality (8.47 ± 1.9 out of 12 maximum points) in SANRA. The minimum score obtained was 6 [38] and the maximum was 12 points [41,51]. Ten (58.82%) of these studies presented high methodological quality (≥8 points), and seven of them had a score lower than 8 (41.18%). Inter-rater agreement was almost perfect (κ = 0.845). See Table 5 for more information.

### 3.4. Evidence Synthesis

Evidence was synthesized into eight categories of evidence. Six categories included reviews with healthy participants, exploring exercise modalities, such as aerobic [20,22,23,32], dynamic resistance [22,23], isometric resistance [23,32], blood flow restriction (BFR) and aerobic exercise [35], BFR and resistance exercise [35], and the association of psychological variables [32] on EIH. Another four categories involved participants with musculoskeletal pain exploring aerobic [20,21,31,34], resistance [20,21,31], and isometric [23,31,33], and the influence of psychological variables [32] on EIH.

Limited evidence was identified for all categories of population, exercise, and influence of psychological variables on EIH, except for the effect of aerobic exercise on healthy participants, presenting a moderate certainty of evidence. See Table 6.

### 3.5. Post Compilation

**Figure 2:**
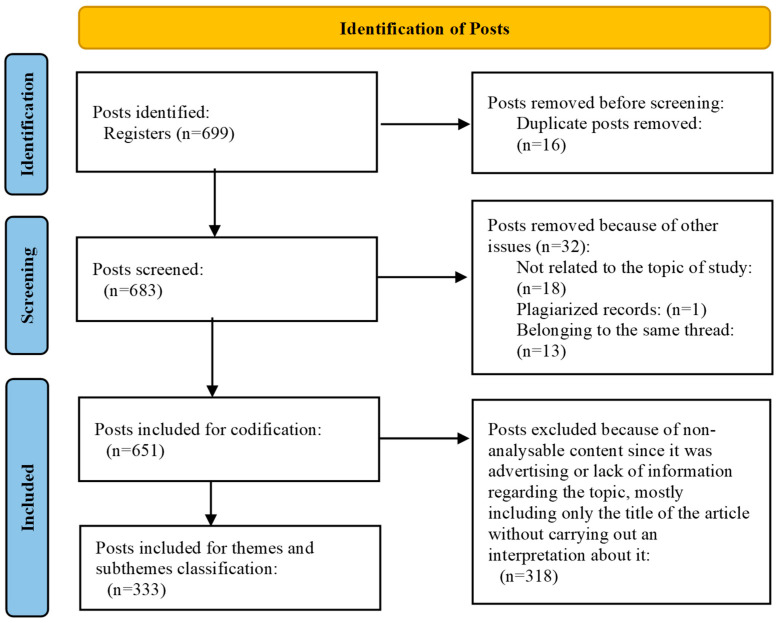
Flowchart of posts’ selection process.

Post identification, screening, and eligibility processes are described in Figure 2. A total of 651 posts were included for code classification; after removing the posts with codes 4 and 6, a total of 333 posts were included for theme and subtheme classification. The collected posts were published between 17 August 2009 and 5 November 2022.

### 3.6. Descriptive Content Analysis

A total of 99 posts were classified into “scientific-related content” (code 1), representing 15.21% of the data. “Scientific quote” (code 2) included 129 (19.82%) posts. “Personal appreciation” (code 3) gathered 43 (6.61%) posts, and “advertising or publicity” (code 4) gathered a total of 16 (2.46%) posts. Only 62 (9.52%) posts were categorized as “misinterpretation of scientific evidence” (code 5). Lastly, “non-analyzable content” (code 6) included 302 (46.39%) posts. The concordance between the reviewers for code classification was substantial, κ = 0.775. Additional data are presented in Table 7.

The user’s profession from the included posts was also explored. Physiotherapists were the professionals who published the greatest number of posts compared with other professionals, with a total of 268 posts (41.17% among all posts). Physiotherapists presented the highest rate of posts throughout all codes, including the “misinterpretation of scientific evidence” (code 5), with 27 (43.55%) posts from physiotherapists among the posts assigned to that code. See Table 7.

### 3.7. Qualitative Content Analysis

For the X content analysis, five themes were established: “exercise effect”, “underlying mechanisms”, “exercise modalities and prescription variables”, “effect location”, and “associated psychosocial variables”. Each was subdivided into subthemes further described in Table 8.

#### 3.7.1. Exercise Effect

This theme was the most frequent, with 211 (57.96%) posts, including four subthemes. The majority (140, 66.35%) of these posts discussed the hypoalgesic effects of exercise in both asymptomatic populations and in patients, where the main theme of posted content concerned the fact that a decrease in pain sensitivity could be elicited by exercise, and the evidence behind it.

“Experimental studies on the effect of exercise have revealed that pain-free individuals show a hypoalgesic response after exercise”.

“OUR LATEST PAPER: #Cycling exercise induces hypoalgesic effects in leg muscles, but not all #pain pathways are affected the same way. Pressure pain thresholds are reduced more than heat pain thresholds. Led by ***** and conducted **** and ****”.

“En pacientes con fibromialgia el efecto hipoalgésico del ejercicio ha sido demostrado en distintos estudios, y el ejercicio de fuerza debidamente individualizado ha mostrado su eficacia y seguridad para reducir el dolor en estos pacientes (****)”.

On the other hand, a significant number of posts (55, 26.10%) highlighted that exercise does not always produce hypoalgesia. Several authors expressed that EIH could be impaired in patients with pain, and thereby, this should not be our only goal when prescribing exercise.

“Our new paper showing Ex induced hypoalgesia (EIH) impaired in WAD with both isometric and aerobic exercise. Moderate physical activity (self-report) may predict EIH but high levels of physical activity may impair EIH ****”.

“Los efectos sobre hipoalgesia inducidos por el ejercicio en pacientes con dolor crónico no son tan buenos como pensamos. Hay otros beneficios de Salud general en los que tendríamos que centrar la intervención. ***** #VCongresoFisioterapiayDolor **** #rompiendomitos”.

A smaller percentage of posts mentioned that exercise can produce hyperalgesia in patients with painful conditions and that professionals should be careful managing this with patients.

“Nice paper addressing exercise induced hypoalgesia in chronic pain (and normals). It’s variable, but exercise may elicit hyperalgesia in those with chronic pain. How to manage that is key https://ncbi.nlm.nih.gov/pubmed/30904519 (accessed on 2 February 2025)”.

Moreover, a few posts emphasized the temporality of the effect beyond the short term, the importance of this, and its possible benefits for some patients.

“Existe fuerte evidencia que muestra no solo los beneficios del ejercicio sobre el dolor percibido en respuesta aguda al ejercicio, sino también como adaptación a largo plazo, lo que lleva a efectos sostenidos de hipoalgesia en adultos sanos que realizan ejercicio con regularidad”.

The posts mostly had a scientific-related profile or were supported by a scientific citation (60.66%). However, approximately 22% of the posts contained misinformation due to a misinterpretation of the evidence, which becomes even more evident in subtheme 1.1, “pain sensitivity effects of exercise in healthy and unhealthy population”, in which most posts from this subtheme (30.71%) were misinterpreted from the scientific evidence.

#### 3.7.2. Underlying Mechanisms

Approximately 5% of the posts discussed the mechanisms underlying EIH. This theme was divided into two subthemes: “opioid hypoalgesic effects” and “non-opioid hypoalgesic effects”. Seventy percent of the posts mentioned the role of non-opioid mechanisms on EIH, which usually refer to cannabinoids and serotonin. Moreover, less than one-third (26.6%) mention both an opioid and non-opioid effect. Most (80%) of the posts had a scientific-related profile or were supported by a scientific citation.

“hasta hace poco se creía que la hipoalgesia del ejercicio era por el sistema endógeno, ahora sabemos que también interviene el sistema cannabinoide #XVIcongresoAEF”.

“Mechanisms of Exercise-Induced Hypoalgesia “suggests involvement of a nonopioid mech in EIH after iso ex”.

#### 3.7.3. Exercise Modalities and Prescription Variables

A large number of these posts (70, 34.29%) referred to the influence of exercise intensity or frequency on hypoalgesia. The authors stated that the greater the intensity or frequency, the greater the hypoalgesic effect.

“El ejercicio induce hipoalgesia. Mas todavía si lo haces durante más tiempo y con mas intensidad. #OpioidesyCanabinoides #NijsFisiocyl”.

“This one found that the hypoalgesic effect of exercise is related to exercise intensity, may also be related to RPE and is affected by the site and type of pain stimulus”.

Controversy was observed in relation to isometric exercise. Two-thirds of the posts commented that isometric exercise causes hypoalgesia, whereas the other third supported the opposite.

“Isometric exercises may be a good entry point for patients with highly provocative tendinopathies, but probably shouldn’t be ‘selling’ them based on exercise-induced hypoalgesia”.

“One bout of exercise reduces pain sensitivity in Parkinson’s. Isometric exercise reduced #Pain more than treadmill exercise. The hypoalgesic effect is systemic (occurs in non-exercised muscles). **** **** **** ****”.

All authors shared that BFR training provided a hypoalgesic effect, with some stating that its effects were greater and lasted longer compared to exercise without BFR. Approximately 30% of these posts mentioned that BFR training caused local and systemic hypoalgesia.

“Aerobic #exercise with blood flow restriction causes local and systemic #hypoalgesia and increases circulating #opioid and #endocannabinoid levels (Hughes et al.)—new in ****”.

“El ejercicio con mayor restricción de flujo sanguíneo, provoca mayor efecto hipoalgésico perdurando más en el tiempo. Aunque la percepción de esfuerzo y el disconfort del paciente son mayores. Importante tenerlo en cuenta en clínica **** #neurorehabLaSalle”.

On the other hand, all the posts referring to aerobic exercise (11.43%) and movement representation methods (8.57%), such as motor imagery or action observation, stated that it produced hypoalgesia.

“Among healthy individuals, the evidence (although of low quality) suggests that aerobic exercise results in a large hypoalgesic effect, whereas resistance training results in only a small effect. Not the narrative that we hear nowadays!”

“La imaginería motora y la observación de acciones son una herramienta alternativa y complementaria al tratamiento de, por ejemplo, pacientes con dolor crónico cervical. Generando hipoalgesia y mejoras en el rango de movimiento”.

Finally, the few posts (4.41%) regarding electrotherapy methods, such as transcranial direct current stimulation (tDCS), supported that it accelerated the hypoalgesic effect of exercise.

“Our latest #publication in the Journal of Pain showing that motor cortex tDCS accelerates the onset of exercise-induced hypoalgesia. Congratulations Jana on publishing your honours work! **** ****”

It should be noted that more than half of the posts mentioned a scientific citation; approximately 18% had a scientific-related profile, and about 17% misinterpreted the evidence.

#### 3.7.4. Effect Location

This theme was addressed in 27 (7.42%) posts, referring to the body location where the pain sensitivity effect is produced after exercise. This pain sensitivity change response can be detected locally, which was mentioned in seven posts. These posts were categorized into the subtheme “local effect of EIH”.

“NEW STUDY: Cycling causes #hypoalgesia of the exercised quadriceps but not of the non-exercised trapezius. Result indicates exercise-induced hypoalgesia is not systemic. Any comments, ****? #medicine #exercise“

When the content was published mentioning a remote effect, which was the case for most publications on this theme (20, 74.07%), it was placed in the subtheme “remote effect of EIH”.

“Jeremy Loenneke **** I was pretty skeptical of the systemic hypoalgesia following exercise (I’m not sure why in retrospect) But we have seen this in our lab as well...even in response to handgrip exercise”

It is important to highlight that in both subthemes, most (23, 85.19%) of the published content was shared by accounts with a scientific-related profile or supported by a scientific citation. In addition, on some occasions, X users incorporated the possibility of the hypoalgesia effect occurring locally and systematically in their posts.

“Got #Pain? The Oxford Journal recently reported that #isometric #exercise produced local & remote hypoalgesia”.

“Yup. Same thing as every exercise. Exercise has local and systemic effects. We build tolerance to activity via hypoalgesia, habituation, systemic anti-inflammatory effects. If a quad extension helps knee pain I think planks can help low back pain”

Associated psychosocial variables

This theme, with 36 (9.90%) posts, corresponded to the variables that have relationships with non-specific responses that can influence EIH. Among them were those linked to the supraspinal mechanism. These posts represented groups related to the scientific area for the most part. Some 91.67%, which corresponded to 33 posts, expressed that “expectations and beliefs are associated with EIH”.

“**** “Las expectativas de si el ejercicio va a producir dolor y la información aportada sobre el mismo influye en la respuesta hipoalgésica” #sefidejercicio22”.

“La hipoalgesia no debería ser el motivo único para hacer ejercicio, debemos tener en cuenta las preferencias del paciente para mejorar los resultados y animar a los sujetos a mover más las zonas no dolorosas #VCongresoFisioterapiaYDolor **** ****”.

“Finally got around to reading this one: Power of Words: Influence of Preexercise Information on Hypoalgesia after Exercise-Randomized Controlled Trial. https://europepmc.org/article/med/32366799…, accessed on 2 February 2025, Major finding here is really the hyperalgesia that occurred in the negative expectations group”.

“The issue covers a wide range of topics, from exercise-induced hypoalgesia to psychologically informed physical therapy”.

The other subtheme, with only three (8.33%) posts, addressing that environmental factors can be associated with the hypoalgesia response, was named “contextual variables and hypoalgesia”.

McKenzie Institute (****) https://buff.ly/2G6QDIv, accessed on 2 February 2025, “This study provides preliminary evidence that psychosocial variables, such as the family environment and mood states, can affect both pain sensitivity and the ability to modulate pain through exercise-induced hypoalgesia”. #NothingInIsolation #NotARecipe.

## 4. Discussion

We conducted an umbrella review on EIH and juxtaposed the findings with public perceptions on X. Through systematic searches and utilizing the PAGAC method, we established a solid scientific evidence base. Our aim was to assess the alignment between the scientific consensus on EIH and its portrayal on X, always ensuring an evidence-based and objective approach.

The PAGAC presented a synthesis of evidence related to the effects of various exercise modalities on healthy individuals and patients with musculoskeletal pain. For healthy individuals, aerobic exercise presented controversy across meta-analyses, with one indicating a favorable effect [23], and the other a similar effect to the controls [22]. Resistance exercise was subanalyzed for isometric and isotonic modalities, observing discrepancies: the isotonic modality presented favorable hypoalgesic effects while the isometric modality presented a similar effect as the controls [23]. The influence of psychological variables in EIH in healthy individuals remained controversial.

Meta-analyses performed on patients with musculoskeletal pain for aerobic, resistance, and isometric exercises reported a similar hypoalgesic effect to the controls. Additionally, as observed with the healthy individuals, the effects of psychological variables were controversial.

The predominant X content focused on exercise and its ability to induce or not induce hypoalgesia in symptomatic and non-symptomatic individuals. Most posts were from healthcare professionals and reflected findings from the scientific literature. EIH was shown to be present in healthy individuals after a single exercise session; however, its occurrence in pain populations is uncertain. Misinformation, especially confusing “hypoalgesia” and “analgesia”, is prevalent and poses a challenge for proper interpretation by users. This misinformation on X can have ethical and professional repercussions, given the high search for medical information online.

### 4.1. Exercise Effect

Based on the data obtained, it appears that content related to exercise and its potential to induce hypoalgesia or not in symptomatic and non-symptomatic individuals is the most common issue addressed in the X community. Most of this content was posted by healthcare professionals, in line with the conclusions extracted from the scientific literature. The results of our analysis show that EIH was shown to be present in healthy individuals after a single bout of exercise, with an effect lasting for 15 to 45 min, presenting contradictory findings regarding exercise protocols [23,33,47,50,53]. However, it was unclear whether EIH occurs in pain populations. Possible explanations arise from the population heterogeneity and the variety of exercise protocols [21,23,31,34,47,48,49]. This conclusion is consistent with the information extracted from the X analysis regarding this topic, indicating that the community on this platform has a moderate to good evidence-based understanding of the subject under study. However, we cannot ignore the fact that an important percentage of posts on this theme misunderstood this same evidence. Most misunderstandings came from mistaking the terms “hypoalgesia” and “analgesia”, thus mixing particularities and statements about them. According to pain researchers, “hypoalgesia” is defined as a decrease in sensitivity to a painful stimulus, such as increased pain thresholds or decreased pain intensity, to a standardized painful stimulus [48]. Hypoalgesia can be studied in both the healthy and pain populations, whereas “analgesia”, which is a reduction in perceived pain intensity, can only be studied in pain populations.

This misleading or confusing health-related information has previously been seen in X [57], and its prevalence could range from 30% to 87% [58]. This is a call to action; users, and even more so, healthcare providers, should be extremely careful with the information posted on social media. Up to 92.6% of patients and healthy individuals seek medical information through the Internet, X being one of the most searched with this aim (82.6%) [59]. Misleading information could cause several ethical problems in terms of patients’ safety and their informed decision-making. Misinformation also affects healthcare providers, reducing trust and credibility in the patient-professional relationship. Professional reputations can also be diminished. Healthcare workers should be aware of the greater impact of posting unreliable information, in terms of wasting public resources.

### 4.2. Underlying Mechanisms

Most mechanisms studied have been based on basic research studies [60,61,62,63], thus, caution is needed when extrapolating this finding to human populations. Moreover, it is unclear whether the mechanisms involved in EIH in healthy individuals are shared or differ from the ones presented in patients with chronic pain.

Central descending opioid and cannabinoid pathways have classically been proposed as the main mechanism of EIH [43,44,48,49,64]. Serotoninergic pathways have also been suggested to be related to EIH [44,48]. The X community agrees with the scientific literature and suggests an influence of opioid and non-opioid mechanisms in EIH. Interaction between pain modulation and cardiovascular response [37], conditioned pain modulation, neuroendocrine stress response [50], and the neuroimmune system have also been suggested as mechanisms related to EIH [48]. Nevertheless, no posts had discussed the role of these mechanisms in EIH.

### 4.3. Exercise Modalities and Prescription Variables

There is controversy regarding the effectiveness of aerobic exercise for inducing EIH [22,23]. Wewege et al. [23] found a moderate significant effect size in favor of this modality, whereas Pacheco-Barrios et al. [22] did not observe an effect. However, the study by Pacheco-Barrios et al. [22] analyzed intragroup differences, which is not recommended [65]. Higher intensity (approximately 75% VO2max) is associated with greater hypoalgesic response; however, it is typically linked with duration, with the combination of intensity and duration being the most important parameter for eliciting hypoalgesia rather than either variable alone [22,23,43]. According to this information, X users support the hypoalgesic intensity-dependent effect of aerobic exercise in healthy individuals. Moreover, lower intensities both associated with or without BFR training could possibly induce EIH; nevertheless, there is scarce evidence to draw a consistent conclusion [35]. X content did not properly discern the effect derived from aerobic or resistance exercise in combination with BFR training. Although BFR with aerobic exercise presented favorable hypoalgesic effects, the systematic review presented scarce evidence supporting this finding. Additionally, controversy appeared for BFR with resistance exercise, given that two studies presented results both favorable and similar to the control. X-related content tended to generalize the favorable effects of BFR with aerobic exercise (derived only from one study), to any kind of exercise in combination with BFR training.

Regarding those with musculoskeletal pain, EIH has been reported for aerobic exercise both after a single bout of exercise and after an exercise protocol intervention of various sessions, with 4–60 minutes’ duration of each session and an incremental intensity of 50–75% VO2max, 40–85% heart rate reserve, or 66–85% maximal heart rate [20,34]. However, aerobic exercise presents uneven results for whiplash-associated disease and for knee osteoarthritis [21,31].

Resistance exercise showed similar results to control interventions in terms of EIH in patients with musculoskeletal pain [20,21,23], independent of isotonic or isometric interventions.

The intensity parameter in a single bout of isometric exercise has shown controversial results for EIH in healthy individuals in both higher and lower intensities (or even lower with BRF training) [22,23,35,51]. However, interventions differ from real practice because these usually consist of several minutes of contraction. It is unclear whether isometric exercise could produce EIH after an exercise protocol for healthy individuals.

For those with musculoskeletal pain, a single bout of any exercise has shown contradictory results, producing no pain sensitivity changes, hypoalgesia, or even hyperalgesia [21,23,31,33]. It is proposed that experiencing pain could influence the pain sensitivity response after a single bout of exercise [23,31,33,41,49]. Effects of isometric exercise protocols in those with musculoskeletal pain have not been studied.

This controversy has also been observed in the X community. Some users indicate that isometric exercise produces hypoalgesia in both healthy individuals and people with pain, whereas others state that this type of exercise does not cause hypoalgesia.

Resistance exercise is the least studied exercise modality in healthy individuals. It appears that EIH could be produced after a single bout of resistance exercise [22,23,41,49]; however, controversial findings have been revealed regarding exercise protocols [47].

For individuals with musculoskeletal pain, a single bout of resistance exercise has shown controversial results related to EIH [21,23,41]. On the other hand, resistance exercise programs after 6 weeks appear to be able to produce EIH in various musculoskeletal pain conditions [20,47]. The hypoalgesic effect after resistance exercise was not discussed on X.

### 4.4. Effect Location

In healthy individuals, a single bout of exercise, regardless of exercise modality, produces EIH local to the exercised area. It is assumed that these effects could also be produced in remote areas but with a lower magnitude [22,23,41,49,51]. Due to lack of research, the effect location after exercise programs is unknown.

Regarding musculoskeletal pain conditions, controversial findings have shown inconsistent responses of local and remote effects after a single bout of exercise [21,23,31,33,34,41]. On the other hand, although the evidence is controversial, exercise programs have been suggested to produce both local and remote hypoalgesic effects [20,21,47].

Although there were few posts on this theme, the majority are in accord and state the local and remote effects of hypoalgesia after exercise.

### 4.5. Associated Psychosocial Variables

Psychosocial factors can play a role in the malfunction or enhancement of EIH. Factors, such as depression, kinesiophobia, and pain catastrophizing, are known to increase pain intensity and reduce nociceptive modulation [66]. However, controversial findings have been reported related to the influence of these factors on EIH response in healthy individuals and pain populations.

In healthy individuals, the correlations between psychological factors and changes in pain thresholds after exercise are inconclusive. Contradictory findings related to catastrophism have been found, given that individuals with high catastrophism experience less or even more EIH [32]. Mood disturbances or fear of pain could be related to lower pain thresholds after exercise. Nevertheless, other studies did not find a correlation between psychosocial factors and changes in pain sensitivity response [32]. Only one study assessed the influence of social factors, such as family environment on EIH in the healthy population, and the authors observed that negative family environments predicted a lower PPT after a single bout of submaximal isometric exercise [67].

In those with musculoskeletal pain, anxiety and kinesiophobia have presented controversial associations with the strength of EIH, whereas depression and catastrophism are not related to EIH at all [32]. The interaction between social factors and EIH has not been explored in patients with musculoskeletal pain.

Overall, results regarding the influence of psychological factors on EIH are mixed. Although the included systematic reviews do not discuss the association of expectations or beliefs with EIH, X users state that positive or negative expectations have an effect in the hypoalgesic response after exercise. Contrary to the scientific literature, the X community also seems to believe that the fear of pain and mood state influence the hypoalgesic response.

### 4.6. Limitations

Primarily, even though there were no explicit language restrictions set, we encountered challenges in interpreting and categorizing posts in non-Latin scripts. This resulted in translation inaccuracies, leading to a subset of posts being labeled as non-analyzable.

Additionally, our classification system permitted posts to be sorted into a maximum of two subthemes. This approach potentially oversimplified the content, given that certain posts might have resonated with multiple subthemes but were restricted to the two deemed most significant. A notable number of posts also contained obsolete links, preventing us from accessing and gleaning further details from these sources.

A significant proportion of the systematic reviews consulted were of suboptimal quality, primarily due to their inclusion of non-controlled and non-randomized trials. Moreover, the methodology of meta-analyses presented limitations, including the grouping of different pain populations, exercise modalities, outcome measures assessed, and body locations. These concerns limited the extrapolation and interpretation of results. Further experimental studies and subsequent meta-analyses must correctly classify population, exercise, and outcome measure parameters to reduce the heterogeneity and enhance the comprehension of these results.

## 5. Conclusions

EIH has been studied across several reviews, exposing significant contributions to this field. However, the certainty of evidence varies significantly based on the exercise modality and population characteristics. The influence of psychological variables on EIH in both healthy individuals and patients with musculoskeletal pain emerges as a promising area for future research.

The included studies exhibit a high average methodological quality and a risk of bias ranging from low to high. The scientific evidence from this umbrella review seemingly indicates the presence of hypoalgesic effects induced by exercise in asymptomatic individuals. However, this effect remains unverified in patients with musculoskeletal pain.

Although the descriptive and qualitative content analysis of posts revealed a significant representation of science-related content and scientific citations, there was a concerning proportion of misinterpretations of scientific evidence. This underscores the imperative for more precise and evidence-based communication on social media platforms. One notable confusion lies in the conflation of exercise-induced hypoalgesic effects with the concept of exercise-induced analgesic effects.

## Figures and Tables

**Table 1:** Characteristics of the included systematic reviews and systematic reviews with meta-analyses.

Study	Review Characteristics				Results Synthesis					
	Studies Included	Aims	Population	Intervention	Population	Exercise Modality	Exercise Doses	Outcome Measure	Summary	Improvement
*Systematic Reviews with Meta-analysis*										
Belavy et al., 2021 [20]	17 Parallel RCTs 1 Cross-over RCT	Study the effectiveness of exercise on peripheral and/or central pain sensitization compared to no exercise, other conservative non-exercise interventions	Population: healthy subjects, patients with fibromyalgia, neck or upper quadrant pain, type 2 diabetes, and Achilles tendon pain Mean age range (years): 25–60 Gender: NI	Exercise interventions: ○ Resistance (isot. and isom.) ○ Aerobic ○ Pilates ○ Stretching ○ Proprioception exercises Comparison group: ○ No intervention ○ Education ○ Hyperbaric oxygen therapy ○ Massage ○ Usual care ○ Stress management ○ Placebo TENS ○ Monthly group meetings ○ Non-exercise-based pool therapy	Msk. pain	Various	Program of 4–16 weeks	Local and remote PPT	Exercise induced greater PPTs compared to no treatment and conservative non-exercise interventions (15 studies; *g =* 0.551; 95%CI: 0.222, 0.879; I^2^ = 80.7%; GRADE: low). Exercise induced greater PPTs compared to only conservative non-exercise interventions (11 studies; *g =* 0.603; 95%CI: 0.159, 1.046; I^2^ = 86.6%; GRADE: low).	Exer. > Cont.
						Aerobic	Program of 6–16 weeks	Local and remote PPT	Exercise and control presented similar changes in PPTs, including in the control, no treatment and conservative non-exercise interventions (5 studies; *g =* 0.695; 95%CI: −0.011, 1.402; I^2^ = 85.1%; GRADE: very low).	Exer. ≈ Cont.
						Resistance	Program of 6–12 weeks	Local and remote PPT	Exercise and control presented similar changes in PPTs, including in the control, no treatment and conservative non-exercise interventions (7 studies; *g =* 0.491; 95%CI: −0.043, 1.024; I^2^ = 84.8%; GRADE: low).	Exer. ≈ Cont.
						Multimodal	Program of 8–16 weeks	Local and remote PPT	Exercise and control presented similar changes in PPTs, including in the control, no treatment and conservative non-exercise interventions (5 studies; *g =* 0.270; 95%CI: −0.019, 0.558; I^2^ = 12.5%; GRADE: low).	Exer. ≈ Cont.
					Fibromyalgia	Several	Program of 4–16 weeks	Local and remote PPT	Exercise induced greater PPTs compared to no treatment and conservative non-exercise interventions (8 studies; *g =* 0.551; 95%CI: 0.098, 1.004; I^2^ = 79.7%; GRADE: very low).	Exer. > Cont.
					Neck or upper quadrant pain	Several	Program of 6–12 weeks	Local and remote PPT	Exercise induced greater PPTs compared to no treatment and conservative non-exercise interventions (5 studies; *g =* 0.666; 95%CI: 0.014, 1.317; I^2^ = 87.3%; GRADE: low).	Exer. > Cont.
								Local PPT	Exercise induced greater PPTs compared to (3 studies; *g =* 0.429; 95%CI: 0.173, 0.686; I^2^ = 0%; GRADE: very low).	Exer. > Cont.
								Remote PPT	Exercise and control presented similar changes in PPTs (3 studies; *g =* 0.245; 95%CI: −0.090, 0.580; I^2^ = 26.0%; GRADE: very low).	Exer. ≈ Cont.
Hall et al., 2020 [21]	10 Parallel RCTs 2 Cross-over studies 2 Quasi-exp. studies 2 Cohorts intervention studies	Explore the effect of exercise on pain processing and motor function in patients with knee OA	Population: patients with knee OA Mean age range (years): 61–69 Gender (M/F): 10–58%/42–90%	Exercise interventions: ○ Single bout of resistance (isotonic and isometric) or aerobic exercise ○ Exercise program (strengthening and/or proprioception) for 5–12 weeks Comparison group: ○ Exercise + another therapy (tDCS, intra-articular injection, neuromuscular electrical stimulation, geotherapy, phototherapy) ○ Non-supervised exercise program ○ Standard treatment ○ Rest	Knee OA	Several	Single bout	Local PPT	Exercise induced greater PPTs than control (5 studies; *g =* 0.26; 95%CI: 0.02, 0.51; I^2^ = 46%; GRADE: very low).	Exer. > Cont.
								Remote PPT	Exercise and control presented similar changes in PPTs (4 studies; *g =* 0.09; 95%CI: −0.11, 0.29; I^2^ = 0%; GRADE: very low).	Exer. ≈ Cont.
							Program of 5–12 weeks	Local PPT	Exercise and control presented similar changes in PPTs (8 studies; *g =* 0.23, 95%CI: −0.01, 0.47; I^2^ = 64%) or remote measures.	Exer. ≈ Cont.
								Local TS	Exercise and control presented similar changes in TS (3 studies; *g =* 0.38; 95%CI: −0.08, 0.85; I^2^ = 77%).	Exer. ≈ Cont.
								Remote PPT	Exercise and control presented similar changes in PPTs (4 studies; *g =* 0.33; 95%CI: −0.13, 0.79; I^2^ = 75%).	Exer. ≈ Cont.
						Aerobic	Single bout	Local PPT	Exercise and control presented similar changes in PPTs (2 studies; *g =* 0.19; 95%CI: −0.07, 0.45; I^2^ = 0%; GRADE: NA).	Exer. ≈ Cont.
								Remote PPT	Exercise and control presented similar changes in PPTs (2 studies; *g =* 0.06; 95%CI: −0.22, 0.34; I^2^ = 13%; GRADE: NA).	Exer. ≈ Cont.
						Resistance	Single bout	Local PPT	Exercise and control presented similar changes in PPTs (5 studies; *g =* 0.23; 95%CI: −0.05, 0.50; I^2^ = 59%; GRADE: NA).	Exer. ≈ Cont.
								Remote PPT	Exercise and control presented similar changes in PPTs (4 studies; *g =* 0.19; 95%CI: −0.01, 0.39; I^2^ = 0%; GRADE: NA).	Exer. ≈ Cont.
Pacheco Barrios et al., 2020 [22]	4 Parallel RCTs 7 Cross-over studies 20 Quasi-exp. studies 4 Cohorts intervention studies	Evaluate PT response to exercise in healthy subjects	Population: healthy subjects Mean age range (years): Unclear. Gender: Unclear	Exercise interventions: ○ Single bout of strength exercise (isot. and isom.) at different intensities ○ Single bout of aerobic exercise at different intensities Comparison group: ○ Deep breathing ○ Rest	Healthy subjects	Several *(across various intensities)*	Single bout	Local and remote PT	Exercise induced greater PTs than control (67 pairwise comparisons; *g =* 0.19, 95%CI: 0.11, 0.27; I^2^ = 7.5%; GRADE: NA).	Exer. > Cont.
						*Several (low intensity)*	Single bout	Local and remote PT	Exercise and control presented similar changes in PTs (7 pairwise comparisons; *g =* −0.10; 95%CI: −0.36, 0.17; I^2^ = 0%; GRADE: NA).	Exer. ≈ Cont.
						*Several (moderate intensity)*	Single bout	Local and remote PT	Exercise and control presented similar changes in PTs (24 pairwise comparisons; *g =* −0.13; 95%CI: 0.00, 0.27; I^2^ = 0%; GRADE: NA).	Exer. ≈ Cont.
						*Several (high intensity)*	Single bout	Local and remote PT	Exercise induced greater PTs than control (36 pairwise comparisons; *g =* −0.27; 95%CI: 0.16, 0.38; I^2^ = 9%; GRADE: NA).	Exer. > Cont.
						Aerobic	Single bout	Local and remote PT	Exercise and control presented similar changes in PTs (30 pairwise comparisons; *g =* 0.05; 95%CI: −0.06, 0.16; I^2^ = 0%; GRADE: NA).	Exer. ≈ Cont.
						Resistance	Single bout	Local and remote PT	Exercise induced greater PTs than control (37 pairwise comparisons; *g =* 0.34; 95%CI: 0.23, 0.44; I^2^ = 0%; GRADE: NA).	Exer. > Cont.
Senarath et al., 2022 [31]	7 Parallel RCTs 3 Cohorts intervention studies 1 Cross-over study	Explore different exercise interventions in EIH in patients with neck pain	Population: individuals with chronic WAD and people with chronic NSNP Mean age range (years): 23.8 to 44.5. Gender (M/F): 29.46%/70.54%	Exercise interventions: ○ Submaximal aerobic exercise ○ Isometric exercise ○ Proprioceptive training ○ Active stretching Comparison group: ○ Passive cervical mobilization ○ Passive scapular correction ○ HVLA manipulation ○ Massage	Neck pain	Resistance (isometric) vs. Aerobic	Single bout	Local PPTs	Isometric exercise presents greater improvements in PPTs compared to aerobic exercise (2 studies; MD = −0.21; 95%CI: −0.43, 0.00; I^2^ = 92%; GRADE: very low).	Resistance > Aerobic
						Resistance (isometric) vs. Aerobic	Single bout	Remote PPTs	Isometric and aerobic exercise presented similar changes in PPTs (2 studies; MD = 0.01; 95%CI: −0.33, 0.35; I^2^ = 0%; GRADE: very low).	Resistance ≈ Aerobic
						Aerobic (local vs. remote)	Single bout	Local vs. remote PPTs	Aerobic exercise induced similar changes in PPTs in local and remote locations (3 studies; MD = −0.01; 95%CI: −0.20, 0.18; I^2^ = 56%; GRADE: very low).	Local ≈ Remote
Wewege et al., 2020 [23]	5 Parallel RCTs 8 Cross-over studies	Explore the effects of exercise in experimentally induced pain in healthy subjects and patients with chronic msk. pain	Population: healthy subjects and patients with chronic msk. pain Mean age range (years): 19–64 in healthy subjects; 49–62 in patients. Gender (M/F): 55%/45% in studies with healthy subjects; 40%/60% in patients	Exercise interventions: ○ Aerobic exercise ○ Isometric exercise ○ Isotonic exercise Comparison group: ○ Rest ○ Sham exercise	Healthy subjects	Aerobic	Single bout	Local and remote PPT, and HPT	Exercise induced greater PTs than control (7 studies; *g =* −0.85; 95%CI: −1.58, −0.13; I^2^ = 99%).	Exer. > Cont.
						Resistance (isotonic)	Single bout	Local PPTs	Exercise induced greater PTs than control (2 studies; *g =* −0.45; 95%CI: −0.69, −0.22; I^2^ = 0%; GRADE: NA).	Exer. > Cont.
						Resistance (isometric)	Single bout	Local PPTs and CPT	Exercise and control presented similar changes in PTs (3 studies; *g =* −0.16; 95%CI: −0.36, 0.05; I^2^ = 98%; GRADE: NA).	Exer. ≈ Cont.
					Chronic msk. pain	Resistance (isotonic)	Single bout	Local PPT	Exercise and control presented similar changes in PTs (1 study; *g =* −0.12; 95%CI: −0.31, 0.07; I^2^ = 95%; GRADE: NA).	Exer. ≈ Cont.
						Resistance (isometric)	Single bout	Local PPT	Exercise and control presented similar changes in PTs (3 studies; *g =* −0.41; 95%CI: −1.08, 0.25; I^2^ = 95%; GRADE: NA).	Exer. ≈ Cont.
***Systematic Reviews***										
Bonello et al., 2021 [33]	3 Parallel RCTs 5 Cross-over studies 3 Cohorts intervention studies 1 Quasi-exp. study	Explore EIH after isometric exercises in patients with msk. pain	Population: patients with msk. pain Mean age range (years): 20.4–65.2. Gender (M/F): 35.4%/63.6%	Exercise interventions: ○ Single session of isometric exercise Comparison group: ○ Aerobic exercise ○ Isotonic exercise ○ Kinesio tape ○ TENS ○ Rest	Msk. pain	Resistance (isometric)	Single bout	Local PTs, Pain tolerance, and CPM	Isometric exercise did not induce EIH consistently for people with msk pain. In contrast with studies in people with widespread pain, such as fibromyalgia, isometric exercise did not generally induce hyperalgesia.	Exer. ≈ Cont.
Karanasios et al., 2023 [35]	2 Parallel RCTs 4 Cross-over studies	Explore EIH after LIE-BFR in healthy subjects and patients	Population: healthy individuals Mean age (years): 24.1. Gender (M/F): 56%/44%	Exercise interventions: ○ Low-intensity aerobic with BFR ○ Low-intensity isotonic resistance exercise with BFR ○ Low-intensity isometric exercise with BFR Comparison group: ○ Low-intensity aerobic or resistance exercise alone ○ High-intensity aerobic or resistance alone ○ Rest	Healthy subjects	Resistance LIE-BFR with low occlusion *(compared to LIE)*	Single bout	Local PPT	Controversial findings appeared with 1 study presenting greater, and 1 similar changes.	Controversy
								Remote PPT	Controversial findings appeared with 1 study presenting greater, and 1 similar changes.	Controversy
						Resistance LIE-BFR with high occlusion *(compared to LIE)*	Single bout	Local PPT	Controversial findings appeared with 1 study presenting greater, and 1 similar changes.	Controversy
								Remote PPT	Controversial findings appeared with 1 study presenting greater, and 1 similar changes.	Controversy
						Aerobic LIE-BFR with light occlusion *(compared to LIE)*	Single bout	Local PPT	Aerobic LIE-BFR with light occlusion induced greater PPTs than LIE (1 study).	Exer. > Cont.
						Aerobic LIE-BFR with high occlusion *(compared to LIE)*	Single bout	Local PPT	Aerobic LIE-BFR with high occlusion induced greater PPTs than LIE (1 study).	Exer. > Cont.
								Remote PPT	Aerobic LIE-BFR with high occlusion induced greater PPTs than LIE (1 study).	Exer. > Cont.
Munneke et al., 2020 [32]	6 Quasi-exp. Studies 2 Cross-sectional studies 1 Cohort intervention study	Explore the association between psychosocial factors and EIH in healthy people and patients with msk. pain	Population: healthy subjects and patients with msk. pain Mean age range: 20.9 to 67.4 in healthy individuals; 44.5 to 52 in pain subjects Gender (M/F): 50%/50% in healthy population; 29.8%/70.2% in people with msk. pain	Intervention group: ○ Single bout of isometric exercise ○ Single bout of aerobic exercise Comparison group: ○ Rest	Healthy subjects	Several	Single bout	Local and remote PPTs, and evoked pressure pain	Most studies (4 out of 5) did not find a significant association between psychological factors (pain catastrophizing, family environment, mood state, anxiety, fear of pain, kinesiophobia) on EIH.	-
					Msk. pain	Several	Single bout	Local and remote PPTs, HPT, and CPT	No significant influence of psychological factors (pain catastrophizing, anxiety, depression, kinesiophobia) on EIH (4 studies).	-
Tan et al., 2022 [34]	4 Parallel RCTs 4 Cohorts intervention studies 3 Quasi-exp. studies	Explore the effects of aerobic exercise on EIH in patients with msk. conditions	Population: patients with msk pain Mean age range (years): 34 to 56 Gender (M/F): 0–62%/38–100%	Intervention group: ○ Single bout of aerobic exercise ○ Exercise program (2 to 12 weeks) of aerobic exercise Comparison group: ○ Conventional physical therapy ○ General health advice ○ Maintain normal activity	Msk. pain	Aerobic	Single bout and Program of 2 weeks	Local and remote PPTs	Exercise induced greater PTTs than control (3 studies).	Exer. > Cont.
					Low back pain	Aerobic	Single bout and Program of 6 weeks	Local and remote PPTs and evoked pain	Exercise induced greater PTTs than control (5 studies).	Exer. > Cont.
					Neck pain	Aerobic	Program of 10–12 weeks	Local and remote PPTs	Exercise induced greater PTTs than control (2 studies).	Exer. > Cont.
					Knee OA	Aerobic	Single bout	Local and remote PPTs	Exercise induced greater PPTs than control (1 study).	Exer. > Cont.

AOP, arterial occlusive pressure; BFR, blood flow restriction; Conc, concentric, Cont, control; CPM, conditioned pain modulation; CPT, cold pain threshold; Ecc, eccentric; EIH: exercise-induced hypoalgesia; Exer, exercise; HIE, high intensity exercise; HPT, heat pain threshold; HVLA, high velocity low amplitude; LIE, low-intensity exercise; NRS, numerical rating scale; NSNP, nonspecific neck pain; OA, osteoarthritis; PPT, pressure pain threshold; Quasi-exp, quasi-experimental; RCT, randomized controlled trial; TENS, transcutaneous electrical nerve stimulation; TS, temporal summation; WAD, whiplash associated disorders; VAS, visual analogue scale.

**Table 2:** Characteristics of the included narrative reviews.

Study	Study Type	Study Objectives	Summary
Koltyn et al., 2000 [36]	Review	To summarize human and animal research about mechanisms responsible of exercise hypoalgesia	In humans, hypoalgesia following exercise has been found using a variety of noxious stimuli and it seems to be most consistent when the exercises are performed at high intensities. EIA has been demonstrated in rodents and both opioid and non-opioid systems appears to be activated.
Koltyn et al., 2002 [13]	Leading Article	To examine if hypoalgesia occurred following exercise and to summarize what intensities of exercise were associated with a hypoalgesic response	Hypoalgesia occurred consistently following high-intensity exercise. EIH was found most consistently with a workload of 200 watts and above.
Kotlyn et al., 2006 [37]	Review	To examine the interaction between pain modulatory and cardiovascular systems during and following exercise	Exercise significantly alters cardiovascular responses and these alterations appear to be associated with changes in pain perception.
Hoffman et al., 2007 [38]	Review	To discuss current understanding of the acute and chronic effects of exercise on mood and pain perception	Aerobic exercise can cause an acute improvement in mood as well as a reduction in the perception of pain from a painful stimulus. Regular exercise training also may offer some protection from depression and chronic pain conditions.
Nijs et al., 2012 [40]	Narrative Review	To review the available evidence addressing the effects of exercise on central pain modulation in patients with chronic pain.	Exercise produces hypoalgesia in healthy individuals, resulting in generalized increased pain tolerance during and immediately following exercise. Aerobic exercise activates pain facilitation in some patients with chronic pain and central sensitization.
Reimers et al., 2012 [39]	Literature Review	To clear whether physical training can induce long-term pain relief in lower back pain, hip and knee osteoarthritis and primary fibromyalgia.	Systematic physical training can have a significant pain-relieving effect on all three pain syndromes, independently on the selected training modality. Strength training, however, was significantly more often effective than aerobic training. The duration both of the weekly training and the training period did not significantly influence the hypoalgesic effect.
Titze et al., 2016 [43]	Focus Review	To highlight the health-promoting but also potentially unfavorable effects of physical activity in healthy people and patients	In healthy volunteers, physical exercise leads to a reduction in pain sensitivity locally and multisegmentally. High-intensity aerobic exercise seems to trigger more robust EIH effects than lower intensity exercise. In contrast, patients may have an hyperalgesic response to exercise due to a reduced endogenous pain inhibition activity.
Ortigosa Cunha et al., 2016 [42]	Review	To discuss the use of EIH as part of chronic pain management	Exercise does not need to be of high-intensity to have an effect on pain management. There is evidence that some groups of chronic pain patients may have the capacity to exercise at intensities and durations that appear to be required to elicit EIH in healthy subjects.
Sluka et al., 2018 [44]	Review	To review the underlying mechanisms of exercise on pain and analgesia.	Regular physical activity produces analgesia through activation of opioids and serotonin, through the increase in anti-inflammatory macrophages and by reducing glial cells activation.
Rice et al., 2019 [48]	Focus article	To provide a contemporary review of the acute effects of exercise on pain and pain sensitivity, including in people with chronic pain conditions	EIH is more variable in chronic pain populations and may be impaired in some people. Interactions between the opioid and endocannabinoid systems and between the opioid and serotonergic systems seem to be important in determining EIH.
Vaegter et al., 2020 [49]	Narrative review	To evaluate the modulatory effects of exercise on pain.	Aerobic and isometric exercise reduces pain sensitivity in both the involved and remote musculature in pain-free subjects. In patients with widespread pain, high pain sensitivity or impaired CPM, both aerobic and isometric exercises may cause a widespread increase in the pain sensitivity.
Vaegter et al., 2020 [49]	Review	To overview the changes in pain perception after acute and regular exercise in pain-free individuals and in individuals with chronic pain, and to discuss the possible underlying mechanisms	In healthy people, a single bout of exercise decreases pain sensitivity. In people with chronic pain, exercise may produce hyperalgesia. The activation of opioid and cannabinoid systems, the release of stress hormones, the activation of baroreceptors or the reduction in central nervous system sensitivity are some mechanisms that could explain EIH.
Leitzelar et al., 2021 [45]	Narrative review	To summarize the preclinical and clinical research examining the effects of exercise on neuropathic pain in different populations	Preclinical evidence states aerobic exercise protocols reduce neuropathic nociception. Clinical evidence points that exercise training reduced sensory (i.e., pain intensity, pain characteristics) or affective (i.e., how troublesome the pain is) components of neuropathic pain.
Kuithan et al., 2022 [46]	Narrative review	To provide an overview of EIH and summary of the underlying mechanisms and mediating factors	EIH seems to be consistent in healthy population, but not in people with chronic pain conditions. Potential mechanisms that may influence EIH include opioids, cannabinoids, serotonin, stress hormones, CPM, cardiovascular changes, or the immune system.
Song et al., 2022 [47]	Narrative review	To provide a contemporary review of EIH, and to discuss potential underlying mechanisms	Training may increase pain tolerance, but not pain threshold in healthy individuals. In patients with chronic pain, a single bout of exercise seems to be insufficient, so that several sessions (training) are necessary to induce these hypoalgesic effects.

CPM, conditioned pain modulation; EIH, exercise-induced hypoalgesia.

**Table 3:** Evaluation of systematic reviews and systematic reviews with meta-analysis with the Modified Quality Assessment Scale for Systematic Reviews.

Studies		Belavy et al., 2021 [20]	Bonello et al., 2021 [33]	Hall et al., 2020 [21]	Karanasios et al. 2023 [35]	Munneke et al., 2020 [32]	Pacheco-Barrios et al., 2020 [22]	Senarath et al., 2022 [31]	Tan et al., 2022 [34]	Wewege et al., 2021 [23]
Were the search methods used to find evidence (original research) on the primary question(s) stated?	Explicitly described to allow replication	Yes	Yes	Yes	Yes	Yes	In Part	Yes	Yes	Yes
Was the search for evidence comprehensive?	Adequate number and range of databases	Yes	Yes	In Part	Yes	Yes	Yes	Yes	Yes	Yes
	Alternative searches	Yes	Yes	Yes	Yes	Yes	Yes	Yes	Yes	Yes
	Adequate range of keywords	Yes	Yes	Yes	Yes	Yes	Yes	Yes	Yes	Yes
	Non-English language studies	Yes	No	Yes	No	No	No	No	No	No
Were the criteria for deciding which studies to include in the overview reported?	Explicitly described to allow replication	Yes	Yes	In Part	Yes	Yes	Yes	No	Yes	Yes
	Excludes reviews that do not adequately address inclusion and exclusion criteria	Yes	Yes	Yes	In Part	Yes	Yes	In Part	In Part	Yes
Was bias in the selection of articles avoided?	Two independent reviewers	Yes	Yes	Yes	Yes	Yes	Yes	Yes	Yes	Yes
Were the criteria used for assessing the quality of included studies reported?	Explicitly described to allow replication	Yes	Yes	Yes	Yes	Yes	Yes	Yes	Yes	Yes
Were the methods used to combine and/or compare the findings of relevant studies appropriate?	Meta-analysis conducted on only homogenous data or limitations to homogeneity discussed	Yes	Yes	Yes	Yes	Yes	Yes	Yes	Yes	In Part
	Confidence intervals/effect sizes reported where possible	Yes	Yes	Yes	Yes	Yes	In part	Yes	In Part	Yes
Were conclusions made by the author(s) appropriate?	Supported by the meta-analysis or other data analysis findings	Yes	Yes	Yes	Yes	Yes	Yes	Yes	In Part	Yes
	Conclusions address levels of evidence for each intervention/comparison	Yes	Yes	Yes	No	Yes	Yes	Yes	Yes	Yes
Total		26	24	22	21	24	22	21	21	23

**Table 4:** Results of risk of bias assessment with the ROBIS tool.

Review	Phase 2				Phase 3
	1. Study Eligibility Criteria	2. Identification and Selection Process	3. Data Collection and Study Appraisal	4. Synthesis and Findings	Risk of Bias in the Review
Pacheco-Barrios et al., 2020 [22]	☹	☹	☹	☹	☹
Hall et al., 2020 [21]	☹	☺	☺	☹	☹
Munneke et al., 2020 [32]	☺	☺	☺	☺	☺
Belavy et al., 2021 [20]	☺	☺	☺	☺	☺
Bonelo et al., 2021 [33]	☹	☹	☺	☺	☹
Wewege et al., 2021 [23]	☺	☹	☺	☺	☺
Tan et al., 2022 [34]	☹	☹	☺	☹	☹
Senarath et al. 2022 [31]	☹	☹	☹	☺	☹
Karanasios et al. 2023 [35]	☹	☺	☺	☺	☺

☺ = Low risk of bias; ☹ = High risk of bias; ? = Unclear risk of bias.

**Table 5:** Methodological quality results from narrative reviews with SANRA.

Studies	Justification of the Article’s Importance for the Readership	Statement of Concrete Aims of Formulation of Questions	Description of the Literature Search	Referencing	Scientific Reasoning	Appropriate Presentation of Data	Total
Koltyn et al., 2000 [36]	1	2	0	2	2	0	7
Koltyn et al., 2002 [13]	2	2	0	1	2	0	7
Koltyn et al., 2006 [37]	2	2	0	2	2	0	8
Hoffman & Hoffman, 2007 [38]	1	1	0	2	2	0	6
Reimers & Reimers, 2012 [39]	2	2	2	2	2	2	12
Nijs et al., 2012 [40]	2	2	2	2	2	1	11
Naugle et al., 2012 [41]	2	2	0	2	2	0	8
Cunha et al., 2016 [42]	1	2	1	2	2	0	8
Titze et al., 2016 [43]	2	2	0	1	2	0	7
Sluka et al., 2018 [44]	1	1	0	2	2	1	7
Rice et al., 2019 [48]	2	2	1	2	2	0	9
Vaegter et al., 2020 [49]	1	2	0	2	2	0	7
Vaegter et al., 2020 [50]	1	2	0	2	2	0	7
Leitzelar et al., 2021 [45]	2	2	1	2	2	0	9
Kuithan et al., 2022 [46]	2	1	0	2	2	1	8
Song et al., 2022 [47]	2	2	2	2	2	1	11
Yamada et al., 2022 [51]	2	2	2	2	2	2	12

Score: No = 0; In part = 1; Yes = 2. There are no established cut-offs for different grades of quality.

**Table 6:** Evidence synthesis through the Physical Activity Guidelines Advisor Committee (PAGAC).

Population	Exercise modality	Criteria					Effect			
		Applicability	Generalizability	Risk of Bias or Study Limitations	Quality and Consistency	Magnitude and Precision of Effect	Effect Size (95% CI)		Evidence	Direction effect
Healthy subjects	Aerobic	Strong	Moderate	Limited	Moderate	Moderate	Hedges’ *g*	0.85 (1.58, 0.13)	Moderate	Controversy
							Hedges’ *g*	0.05 (−0.06, 0.16)		
Healthy subjects	Resistance (isotonic)	Strong	Moderate	Limited	Moderate	Limited	Hedges’ *g*	0.45 (0.22, 0.69)	Limited	Isot > Con
Healthy subjects	Resistance (Isometric)	Limited	Moderated	Limited	Limited	Not assignable	Hedges’ *g*	0.16 (−0.05, 0.35)	Limited	Isom ≈ Con
Healthy subjects	BFR + Aerobic	Limited	Limited	Limited	Not assignable	Not assignable	-	-	Limited	BFR + Aer > Aer
Healthy subjects	BFR + Resistance	Limited	Limited	Limited	Not assignable	Not assignable	-	-	Limited	Controversy
Healthy subjects	Psychosocial variables	Limited	Limited	Limited	Not assignable	Not assignable	-	-	Limited	Controversy
Msk pain patients	Aerobic	Limited	Limited	Limited	Not assignable	Not assignable	Hedges’ *g*	Local: 0.19 (−0.07, 0.45)	Limited	Aer ≈ Con
							Hedges’ *g*	Remote: 0.06 (−0.22, 0.34)		
							Hedges’ *g*	Local & remote: 0.70 (−0.01, 1.4)		
Msk pain patients	Resistance	Moderate	Limited	Limited	Limited	Limited	Hedges’ *g*	Local: 0.23 (−0.05, 0.5)	Limited	Res ≈ Con
							Hedges’ *g*	Remote: 0.19 (−0.01, 0.39)		
							Hedges’ *g*	Local & remote: 0.49 (−0.04, 1.02)		
Msk pain patients	Isometric	Limited	Limited	Limited	Limited	Limited	Hedges’ *g*	0.41 (−0.25, 1.08)	Limited	Isom ≈ Con
Msk pain patients	Psychological variables	Limited	Limited	Limited	Limited	Not assignable	-	-	Limited	Controversy

Aer, aerobic; Con, control; Isom, isometric; Isot, isotonic; Res, resistance; Msk, musculoskeletal.

**Table 7:** Assignment of codes and user’s profession classification summary.

Code	Definition	Posts (%)	User’s Profession or Account Type	Posts (%)
**Scientific-related content** (Code 1)	Information based on scientific evidence, but does not provide scientific citation	99 (29.73%)	Physiotherapist	63 (63.63%)
			Physician	2 (2.02%)
			Chiropractic	2 (2.02%)
			Other non-healthcare professionals	3 (3.03%)
			Non-identifiable	12 (12.12%)
			Dissemination account	3 (3.03%)
			Sports professional	3 (3.03%)
			Institution	8 (8.08%)
			Other healthcare professional	1 (1.01%)
			Exercise physiologist	2 (2.02%)
**Scientific citation** (Code 2)	Correct content supported by a scientific citation	129 (38.74%)	Physiotherapist	57 (44.19%)
			Chiropractic	4 (3.10%)
			Non-identifiable	24 (18.60%)
			Dissemination account	8 (6.20%)
			Sports professional	5 (3.88%)
			Institution	19 (14.73%)
			Physician	5 (3.88%)
			Other healthcare professionals	2 (1.55%)
			Exercise physiologist	5 (3.88%)
**Personal appreciation** (Code 3)	Opinion without scientific support	43 (12.91%)	Physiotherapist	31 (72.10%)
			Chiropractic	1 (2.33%)
			Non-identifiable	6 (13.95%)
			Institution	1 (2.33%)
			Physician	2 (4.65%)
			Exercise physiologist	1 (2.33%)
			Other healthcare professionals	1 (2.33%)
**Misinterpretation of scientific evidence** (code 5)	Misinformation or content contrary to the scientific literature	62 (18.62%)	Physiotherapist	27 (43.55%)
			Chiropractic	1 (1.61%)
			Non-identifiable	13 (20.97%)
			Dissemination account	4 (6.45%)
			Sports professional	2 (3.23%)
			Institution	3 (4.84%)
			Physician	5 (8.10%)
			Exercise physiologist	2 (3.23%)
			Other healthcare professionals	1 (1.61%)
			Other non-healthcare professionals	4 (6.45%)

**Table 8:** Assignment of themes and subthemes classification summary.

Theme	Posts (%)	Subtheme	Posts (%)	Code	Posts (%)
**1. Exercise effect**	211 (57.97%)	1.1 Pain sensitivity effect of exercise in asymptomatic population and patients	140 (66.35%)	1. Scientific-related content	38 (27.14%)
				2. Scientific citation	34 (24.29%)
				3. Personal appreciation	25 (17.86%)
				5. Misinterpretation of scientific evidence	43 (30.71%)
		1.2 Exercise does not always produce hypoalgesia.	55 (26.10%)	1. Scientific-related content	21 (38.18%)
				2. Scientific citation	22 (40%)
				3. Personal appreciation	9 (16.36%)
				5. Misinterpretation of scientific evidence	3 (5.45%)
		1.3 Exercise may produce hyperalgesia	11 (5.21%)	1. Scientific-related content	4 (36.36%)
				2. Scientific citation	6 (54.55%)
				3. Personal appreciation	1 (9.10%)
		1.4 Timing of the effect	5 (2.37%)	1. Scientific-related content	2 (40%)
				2. Scientific citation	1 (20%)
				3. Personal appreciation	1 (20%)
				5. Misinterpretation of scientific evidence	1 (20%)
**2. Underlying mechanisms**	20 (5.50%)	2.1 Opioid hypoalgesic effects	6 (30%)	1. Scientific-related content	3 (50%)
				2. Scientific citation	2 (33.33%)
				3. Personal appreciation	1 (16.67%)
		2.2 Non-opioid hypoalgesic effects	14 (70%)	1. Scientific-related content	4 (28.57%)
				2. Scientific citation	7 (50%)
				3. Personal appreciation	1 (7.14%)
				5. Misinterpretation of scientific evidence	2 (14.29%)
**3. Exercise modalities and prescription variables**	70 (19.23%)	3.1 EIH with BFR	13 (18.58%)	1. Scientific-related content	1 (7.69%)
				2. Scientific citation	9 (69.23%)
				3. Personal appreciation	1 (7.69%)
				5. Misinterpretation of scientific evidence	2 (15.38%)
		3.2 Cardiovascular EIH	8 (11.43%)	1. Scientific-related content	1 (12.5%)
				2. Scientific citation	5 (62.5%)
				5. Misinterpretation of scientific evidence	2 (25%)
		3.3 Isometric exercise-induced hypolgesia	16 (22.86%)	1. Scientific-related content	3 (18.75%)
				2. Scientific citation	10 (62.5%)
				3. Personal appreciation	1 (6.25%)
				5. Misinterpretation of scientific evidence	2 (12.5%)
		3.4 Influence of exercise intensity on EIH	24 (34.29%)	1. Scientific-related content	7 (29.17%)
				2. Scientific citation	11 (45.83%)
				3. Personal appreciation	1 (4.17%)
				5. Misinterpretation of scientific evidence	5 (20.83%)
		3.5 Movement representation methods and hypoalgesia	6 (8.57%)	1. Scientific-related content	1 (16.67%)
				2. Scientific citation	3 (50%)
				3. Personal appreciation	1 (16.67%)
				5. Misinterpretation of scientific evidence	1 (16.67%)
		3.6 Electrotherapy agents and hypoalgesia	3 (4.29%)	2. Scientific citation	2 (66.67%)
				3. Personal appreciation	1 (33.33%)
**4. Effect location**	27 (7.42%)	4.1 Local effect of EIH	7 (25.93%)	1. Scientific-related content	3 (42.86%)
				2. Scientific citation	3 (42.86%)
				3. Personal appreciation	1 (14.29%)
		4.2 Remote effect of EIH	20 (74.10%)	1. Scientific-related content	7 (35%)
				2. Scientific citation	10 (50%)
				3. Personal appreciation	3 (15%)
**5. Associated psychosocial variables**	36 (9.90%)	5.1 Expectations and beliefs influence EIH	33 (91.67%)	1. Scientific-related content	17 (51.51%)
				2. Scientific citation	13 (39.39%)
				3. Personal appreciation	1 (3.03%)
				5. Misinterpretation of scientific evidence	2 (6.06%)
		5.2 Contextual variables and hypoalgesia	3 (8.33%)	1. Scientific-related content	1 (33.33%)
				2. Scientific citation	2 (66.67%)

BFR, blood flow restriction training; EIH, exercise-induced hypoalgesia.

## Data Availability

Not applicable.

## Supplementary Materials

The following supporting information can be downloaded at https://www.mdpi.com/article/10.3390/medicina61030401/s1, Supplementary Material S1: Search and selection process. Search engines, databases and search equations.
