# Supplementary material for: Understanding Exercise-Induced Hypoalgesia: An Umbrella Review of Scientific Evidence and Qualitative Content Analysis
Source: Medicina (Kaunas). 2025 Feb 25;61(3):401. doi: 10.3390/medicina61030401 (PMC11944103; doi:10.3390/medicina61030401)
Supplement: Supplementary file 1 [file medicina-61-00401-s001.zip › medicina-3485512-supplementary.pdf]

Supplementary Material S1. Search and selection process. Search engines, databases and search equations.

**Search Engine**	**Databases**	**Search (nº)**	**Equation**	**Date**	**Registries (n)**
Scientific literature searches					
PubMed	MEDLINE	Nº1	(“exercise”[MeSH Terms] OR “isometric*”[Title/Abstract] OR “exercis*”[Title/Abstract] OR “exercise therapy”[MeSH Terms] OR (“strength*”[Title/Abstract] OR “resistance exercise”[Title/Abstract]) OR “aerobic exercise”[Title/Abstract]) AND (“pain threshold”[Title/Abstract] OR “hypoalges*”[Title/Abstract] OR “quantitative sensory testing”[Title/Abstract] OR “conditioned pain modulation”[Title/Abstract] OR “temporal summation”[Title/Abstract] OR “wind up”[Title/Abstract] OR “pain perception”[Title/Abstract] OR “somatosensory perception”[Title/Abstract] OR “pressure pain threshold”[Title/Abstract])	23 Nov 2022	189
Web of Science	WOS Core Collection<br>Current Contents Connect<br>Derwent Innovations Index<br>KCI-Korean Journal Database<br>MEDLINE<br>SciELO Citation Index.	Nº1	TS=((“exercise” OR “exercise therapy” OR “isometric” OR (“strengthening” OR “resistance exercise”) OR “aerobic exercise”) AND (“pain threshold” OR “hypoalgesia” OR “quantitative sensory testing” OR “conditioned pain modulation” OR “temporal summation” OR “wind up” OR “pain perception” OR “somatosensory perception” OR “pressure pain threshold”))<br>*Filter:* Review article	23 Nov 2022	298
SciELO	-	Nº1	((exercise) OR (exercise therapy) OR (strengthening) OR (resistance exercise) OR (aerobic exercise)) AND ((pain threshold) OR (hypoalgesia) OR (quantitative sensory testing) OR (conditioned pain modulation) OR (temporal summation) OR (wind up) OR (pain perception) OR (somatosensory perception) OR (pressure pain threshold))	23 Nov 2022	1
PEDro	-	Nº1	*Abstract & Title:* hypoalgesia exercise<br>*Method:* Systematic review	23 Nov 2022	3
		Nº2	*Abstract & Title:* “pain sensitivity” exercise<br>*Method:* Systematic review	23 Nov 2022	4
Google Scholar	-	Nº1	allintitle: exercise hipoalgesia	23 Nov 2022	16
Tweet searches					
Twitter search bar	Twitter	Nº1	“hypoalgesia” (“exercise” OR “physical activity” OR “sport” OR “training” OR “movement”)	22 Nov 2022	504

	Nº2	“hypoalgesic effect” (“exercise” OR “physical activity” OR “sport” OR “training” OR “movement”)	22 Nov 2022	23
	Nº3	“hypoalgesic effects” (“exercise” OR “physical activity” OR “sport” OR “training” OR “movement”)	22 Nov 2022	34
	Nº4	“hypoalgesic response” (“exercise” OR “physical activity” OR “sport” OR “training” OR “movement”)	22 Nov 2022	10
	Nº5	“hypoalgesic responses” (“exercise” OR “physical activity” OR “sport” OR “training” OR “movement”)	22 Nov 2022	1
	Nº6	“hipoalgesia” (“ejercicio” OR “actividad física” OR “deporte” OR “entrenamiento” OR movimiento”)	22 Nov 2022	92
	Nº7	“efecto hipoalgésico” (“ejercicio” OR “actividad física” OR “deporte” OR “entrenamiento” OR movimiento”)	22 Nov 2022	16
	Nº8	“efectos hipoalgésicos” (“ejercicio” OR “actividad física” OR “deporte” OR “entrenamiento” OR movimiento”)	22 Nov 2022	11
	Nº9	“respuesta hipoalgésica” (“ejercicio” OR “actividad física” OR “deporte” OR “entrenamiento” OR movimiento”)	22 Nov 2022	4
	Nº10	“respuestas hipoalgésicas” (“ejercicio” OR “actividad física” OR “deporte” OR “entrenamiento” OR movimiento”)	22 Nov 2022	0
	Nº11	“hypoalgesia” (“ejercicio” OR “actividad física” OR “deporte” OR “entrenamiento” OR movimiento”)	22 Nov 2022	4
	Nº12	“hypoalgesic effect” (“ejercicio” OR “actividad física” OR “deporte” OR “entrenamiento” OR movimiento”)	22 Nov 2022	0
	Nº13	“hypoalgesic effects” (“ejercicio” OR “actividad física” OR “deporte” OR “entrenamiento” OR movimiento”)	22 Nov 2022	0
	Nº14	“hypoalgesic response” (“ejercicio” OR “actividad física” OR “deporte” OR “entrenamiento” OR movimiento”)	22 Nov 2022	0
	Nº15	“hypoalgesic responses” (“ejercicio” OR “actividad física” OR “deporte” OR “entrenamiento” OR movimiento”)	22 Nov 2022	0
